# Online Learning Among Older Adults: Learning Efficacy as a Key Predictor for Mental Health

**DOI:** 10.1093/geroni/igaf122.2951

**Published:** 2025-12-31

**Authors:** Junhua Xiao, Huamao Peng

**Affiliations:** Beijing Normal University, Beijing, Beijing, China (People’s Republic); Beijing Normal University Institute of Developmental Psychology, Beijing, Beijing, China (People’s Republic)

## Abstract

More than 50% of older adults in China now use the Internet, making online learning a key approach for lifelong learning. With age, older adults tend to prioritize positive emotional experiences, indicating that online learning experiences may have a more direct relationship with mental health than learning performance. This study examines whether learning performance influences mental health through learning experiences, particularly focusing on the effect of online learning efficacy, as it aligns with higher psychological needs of older adults. A total of 998 community-dwelling older adults in China (age = 68.50 ± 5.37 years) completed questionnaires assessing self-rated learning performance, learning experiences (via the Online Learning Efficacy Scale), and mental health (via the Geriatric Depression Scale, Social Participation Scale, Subjective Cognitive Decline Scale). Hierarchical regression and mediation analyses were conducted. The results showed that sixty percent of older adults spent more than 1 hour daily on online learning. Learning performance negatively predicted depression, subjective cognitive decline, social participation with demographic variables controlled. After adding the online learning efficacy into the regression model, the regression model’s explanatory power significantly increased. Further analysis showed that online learning efficacy mediated the relationship between learning performance and mental health, with the strongest mediated effect observed on social participation. These findings suggest that for older adults, online learning efficacy is closely linked to their mental health, particularly for social health. Educational programs should prioritize fostering older adults’ sense of competence to promote mental health and positive ageing.

